# Pyoderma gangrenosum und Spinnenbisse: eine Fallserie

**DOI:** 10.1111/ddg.15731_g

**Published:** 2025-08-11

**Authors:** Luca Rapparini, Michelangelo La Placa, Giorgio De Benedetto, Yuri Merli, Annalucia Virdi, Cosimo Misciali, Federico Bardazzi

**Affiliations:** ^1^ Dermatology Unit IRCCS Azienda Ospedaliero‐Universitaria di Bologna Bologna Italy; ^2^ Department of Medical and Surgical Sciences Alma Mater Studiorum University of Bologna Bologna Italy

**Keywords:** Geschwür, Pyoderma gangrenosum, Spinnenbiss, Pyoderma gangrenosum, spider bite, ulcer

Sehr geehrte Herausgeber,

Pyoderma gangrenosum (PG) ist eine seltene entzündliche Hauterkrankung aus der Gruppe der neutrophilen Dermatosen. Klinisch weist es schmerzhafte, unregelmäßige, livid‐erythematöse Hautulzerationen mit unterminierten Rändern auf.^1^ Die Ätiologie des PG ist noch unklar und seine Pathogenese ist noch nicht vollständig verstanden. Die häufige Assoziation mit systemischen Erkrankungen, wie chronisch entzündlichen Darmerkrankungen, rheumatoider Arthritis und hämatologischen Erkrankungen, lässt jedoch auf eine zugrundeliegende immunologische Anomalie schließen; dennoch werden 25–50% der Fälle als idiopathisch eingestuft.^2^, ^3^ Charakteristisch für das PG ist das Pathergie‐Phänomen, also das Auftreten neuer Läsionen oder die Verschlimmerung bereits bestehender Läsionen als Folge kleinerer Traumata, chirurgischer Eingriffe, Injektionen oder Pricktests.^2^, ^4^ In diesem Zusammenhang sind Spinnenbisse interessante, aber wenig bekannte Auslösefaktoren. Obwohl es relativ viel Literatur über PG gibt, haben nur wenige Studien eine mögliche ätiologische Rolle untersucht.^3^, ^5^, ^6^

In Italien sind Spinnenbisse selten und verursachen in der Regel lokale Erytheme, Ödeme, Juckreiz oder Schmerzen, die in der Regel innerhalb weniger Tage ohne Behandlung abklingen. Einige gefährliche Arten, darunter die Europäische oder Mediterrane Schwarze Witwe (*Latrodectus tredecimguttatus*) und die Braune Violinspinne (*Loxosceles rufescens*), können jedoch schwere gesundheitliche Probleme verursachen.^7^

Wir sammelten fünf Fälle von PG, die nach Spinnenbissen bei Patienten auftraten, die zwischen 2023 und 2024 die dermatologische Abteilung der Universität Bologna aufsuchten. Die Patienten wurden über die Verwendung ihrer klinischen Daten in Übereinstimmung mit den Grundsätzen der Deklaration von Helsinki und über die Verwendung von Fotos zu Veröffentlichungszwecken informiert. Alle Patienten wiesen ulzerative Läsionen auf, die zunächst eine nekrotisierende bakterielle Infektion simulierten und wurden mit Antibiotika (Amoxicillin/Clavulansäure oder Azithromycin) und Antiseptika wie Povidon‐Jod behandelt. Bei allen Patienten blieb die Behandlung unwirksam, was die Diagnose PG bestätigte. Die Patienten zeigten Geschwüre von 1,5 bis 15 cm Größe, oft mit unregelmäßigen Rändern, Fibrin und hypertrophem Wundgrund. Die umgebende Haut war häufig erythematös oder purpuriform (Abbildung 1a‐e). Die Lokalisation war überwiegend an den unteren Extremitäten, in einem Fall auch am Bauch. Histologische Untersuchungen erhärteten die Diagnose (Abbildung 1f). Behandelt wurde hauptsächlich mit topischem Clobetasolpropionat unter Okklusion. In einigen Fällen waren zusätzlich systemische Immunsuppressiva wie Cyclosporin oder Methylprednisolon erforderlich. Die Heilungszeiten reichten von 6 bis 22 Wochen, wobei die Hyperchromie bestehen blieb. Die klinischen Merkmale sind in Tabelle 1 zusammengefasst.

**ABBILDUNG 1:**
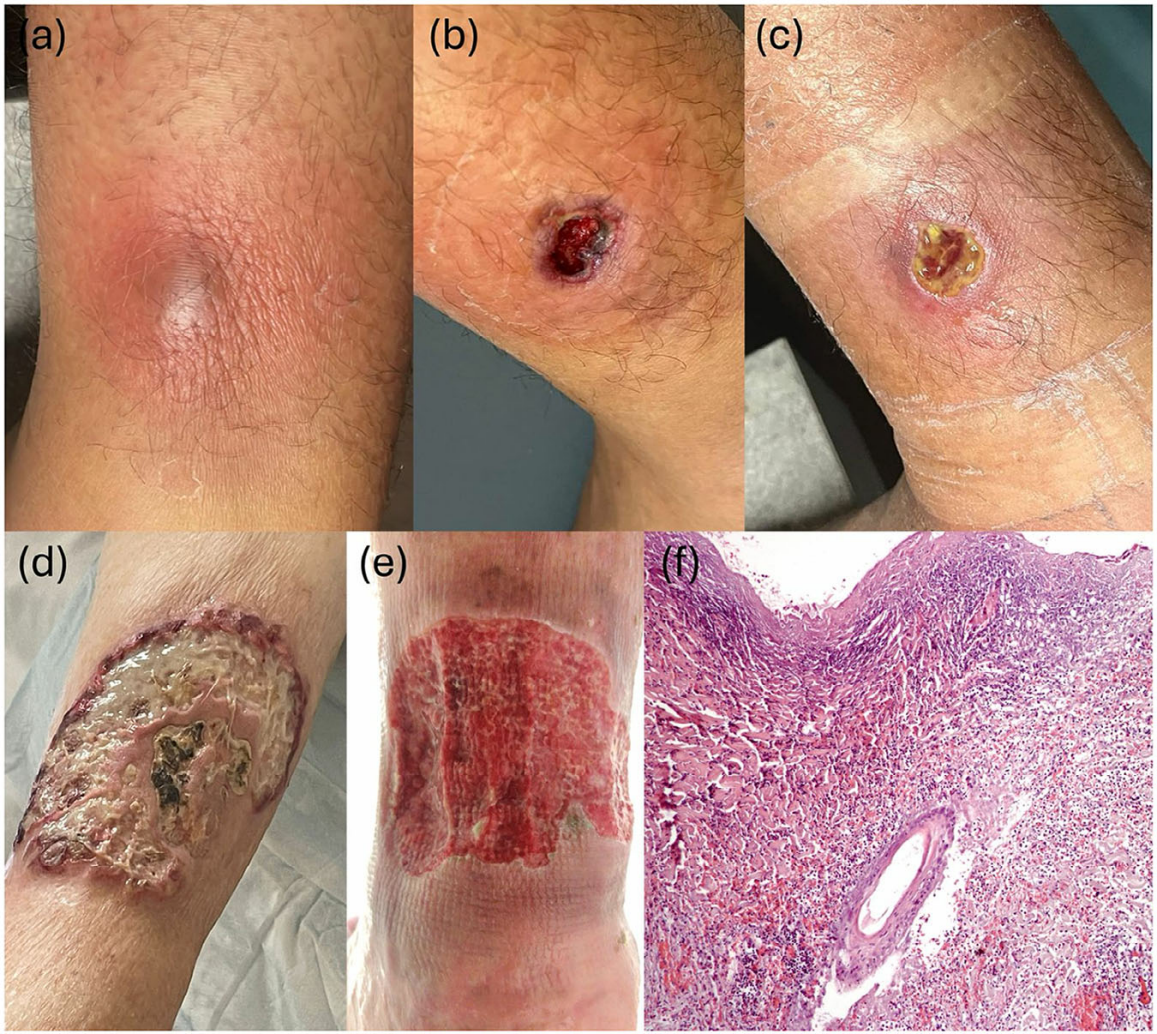
Entwicklung der Läsion bei Patient 1. (a) Vier Tage nach dem Spinnenbiss. (b) Diagnose des Pyoderma gangrenosum (PG) am 13. Tag. (c) Fortschreitende Abheilung der Läsion am 30. Tag. (d) Präsentation bei der PG‐Diagnose von Patient 2 und (e) Patient 4. (f) Die histologische Untersuchung des Biopsats von Patient 2 zeigt ein dichtes und diffuses Infiltrat von Neutrophilen, das sich über die gesamte Dermis erstreckt, Degeneration von Kollagen in der Dermis, Follikulitis und Perifollikulitis sowie Abszessbildung mit Ruptur des follikulären Infundibulums (Hämatoxylin‐Eosin‐Färbung, Originalvergrößerung x 10).

**TABELLE 1:** Merkmale von Patienten mit Pyoderma gangrenosum nach einem Spinnenbiss.

Punkt.	Geschlecht, Alter (Jahre)	Frühere Begleiterkrankungen	Merkmale der Läsion	Behandlungsschema nach Spinnenbiss	Auftreten des PG (Tage) nach Spinnenbiss	Behandlungsschema für PG	PG‐Heilungszeit (Tage)
1	M, 33	Kutane Psoriasis bei Brodalumab‐Therapie	rundes Ulcus von 2 cm Durchmesser mit hypertropher Basis und fibrösem Belag, lateral am linken Bein	Azithromycin 500 mg, für 3 Tage; Fusidinsäure + Betamethason‐Creme, einmal täglich	13	Clobetasolpropionat‐Creme unter Okklusion, einmal täglich	70
2	F, 84	Arterielle Hypertonie, früherer Myokardinfarkt, Divertikulose	15 × 10 cm großes Ulcus am rechten Bein, fibrinöser und granulierender Grund, kleiner nekrotischer Schorf, erythematös‐purpuriforme periläsionale Haut	Amoxicillin/Clavulansäure 875/125 mg Kapseln, dreimal täglich für 14 Tage	20	Clobetasolpropionat‐Creme unter Okklusion, einmal täglich	82
3	F, 53	Hashimoto‐Thyreoiditis bei Ersatztherapie, seronegative Arthritis unter NSAIDs, Fibromyalgie	maximal 7,5 cm durchmessende Plaque mit multiplen Ulzerationen und unscharfen Rändern, periläsional erythematös‐purpuriforme Haut; lateral supramalleolär am linken Bein	Amoxicillin/Clavulansäure 875/125 mg Kapseln, dreimal täglich für 8 Tage	32	Methylprednisolon 16 mg: 2 Kapseln täglich für 7 Tage, dann 1,5 Kapseln täglich für 7 Tage, dann 1 Kapsel täglich für 7 Tage, dann 0,5 Kapseln täglich für 1 Monat, gefolgt von 0,25 Kapseln täglich für 1 Monat	62
4	M, 55	Leichte kutane Psoriasis unter topischer Therapie	unregelmäßig ovales Ulcus, 5 cm Durchmesser, hypertrophe Basis und fibröser Belag, hinterer Aspekt des linken Knöchels	Povidon‐Jod‐Gaze + Elastokompressionsverband	25	Clobetasolpropionat‐Salbe unter Okklusion, einmal täglich; Cyclosporin 100 mg Kapseln, zweimal täglich	160
5	M, 24	Keine	Ovales Ulcus, 1,5 cm durchmessend, hypertrophe Basis, an der rechten Bauchseite	Povidon‐Jod‐Gaze	37	Clobetasolpropionat‐Creme unter Okklusion, einmal täglich	40

*Abkürzung*: PG, Pyoderma gangrenosum

Pyoderma gangrenosum ist eine häufig fehldiagnostizierte Erkrankung, insbesondere in der Anfangsphase. Unserer Erfahrung nach wurden die Patienten zunächst wegen bakterieller Infektionen behandelt. Diese Diagnoseverzögerung unterstreicht, das Ärzte bei der Differenzialdiagnose ulzerativer Läsionen, die auf konventionelle Therapie nicht ansprechen, stärker im Hinblick auf PG sensibilisiert sein sollten.^4^, ^8^

Der mögliche Zusammenhang zwischen Spinnenbissen und PG wirft wichtige Fragen auf: Das lokale Trauma und die akute Entzündung durch das Gift könnten eine abnorme Immunreaktion auslösen (Pathergie‐Phänomen). Ein Trauma ist ein bekannter Stimulus für die Freisetzung von Zytokinen und schadenassoziierten molekularen Mustern (*damage‐associated molecular patterns*), die angeborene Immunreaktionen verstärken. Traumata lösen die Freisetzung von Interleukin (IL)‐8 und IL‐36 aus Keratinozyten aus, beides mutmaßlich PG‐treibende Zytokine. Gewebsverletzungen können auch die Freisetzung von Autoantigenen fördern. Diese Prozesse können ausreichen, um PG auszulösen, insbesondere bei Personen mit pathogenen Varianten in inflammasomassoziierten Genen.^9^ Es kann angenommen werden, dass ein leichtes Trauma durch einen Spinnenbiss in Verbindung mit der Freisetzung von Toxinen und Enzymen im Spinnengift die Aktivierung derselben Kaskade auslöst. Es wäre interessant zu untersuchen, ob es Toxine oder Proteine im Spinnengift gibt, die Entzündungsreaktionen bei genetisch oder immunologisch prädisponierten Personen verstärken können.

Zusammenfassend sind wir der Meinung, dass bei PG verschiedene mögliche Ursachen in Betracht gezogen werden sollten, darunter auch Spinnenbisse als seltener, aber möglicher auslösender Faktor.^10^

## DANKSAGUNG

Open access publishing facilitated by Universita degli Studi di Bologna, as part of the Wiley ‐ CRUI‐CARE agreement.

## INTERESSENKONFLIKT

Keiner.
